# Pharmacological cognitive enhancement—how neuroscientific research could advance ethical debate

**DOI:** 10.3389/fnsys.2014.00107

**Published:** 2014-06-11

**Authors:** Hannah Maslen, Nadira Faulmüller, Julian Savulescu

**Affiliations:** ^1^Oxford Martin School, University of OxfordOxford, UK; ^2^Department of Experimental Psychology, University of OxfordOxford, UK; ^3^Department Values, Technology and Innovation, Delft University of TechnologyDelft, Netherlands; ^4^Oxford Uehiro Centre for Practical Ethics, University of OxfordOxford, UK

**Keywords:** cognitive enhancement, brain function augmentation, ethics, modafinil, ritalin, justice, cheating, personalized enhancement

## Abstract

There are numerous ways people can improve their cognitive capacities: good nutrition and regular exercise can produce long-term improvements across many cognitive domains, whilst commonplace stimulants such as coffee temporarily boost levels of alertness and concentration. Effects like these have been well-documented in the medical literature and they raise few (if any) ethical issues. More recently, however, clinical research has shown that the off-label use of some pharmaceuticals can, under certain conditions, have modest cognition-improving effects. Substances such as methylphenidate and modafinil can improve capacities such as working memory and concentration in some healthy individuals. Unlike their more mundane predecessors, these methods of “cognitive enhancement” are thought to raise a multitude of ethical issues. This paper presents the six principal ethical issues raised in relation to pharmacological cognitive enhancers (PCEs)—issues such as whether: (1) the medical safety-profile of PCEs justifies restricting or permitting their elective or required use; (2) the enhanced mind can be an “authentic” mind; (3) individuals might be coerced into using PCEs; (4), there is a meaningful distinction to be made between the treatment vs. enhancement effect of the same PCE; (5) unequal access to PCEs would have implications for distributive justice; and (6) PCE use constitutes cheating in competitive contexts. In reviewing the six principal issues, the paper discusses how neuroscientific research might help advance the ethical debate. In particular, the paper presents new arguments about the contribution neuroscience could make to debates about justice, fairness, and cheating, ultimately concluding that neuroscientific research into “personalized enhancement” will be essential if policy is to be truly informed and ethical. We propose an “ethical agenda” for neuroscientific research into PCEs.

## Introduction

Recent research in neuroscience and pharmacology has demonstrated that various pharmaceuticals can have modest cognition-enhancing effects in healthy individuals (for reviews, see Repantis et al., 2010; Husain and Mehta, 2011). For example, some studies have shown that modafinil—originally developed for the treatment of narcolepsy—can improve various dimensions of cognitive function in sleep-deprived (Wesensten et al., 2005; Thomas and Kwong, 2006) and non-sleep-deprived healthy adults (Turner et al., 2003; Müller et al., 2004). Similarly, methylphenidate—originally developed for the treatment of Attention Deficit Hyperactivity Disorder (ADHD)—has been shown to improve spatial working memory and planning in healthy adults (Elliott et al., 1997; Mehta et al., 2000).

Unlike the more mundane methods for improving cognitive function—such as exercise and good nutrition (Dresler et al., 2012)—these pharmaceutical cognitive enhancers (PCEs) are thought to raise a host of ethical issues for individuals and society (Greely et al., 2008; Bostrom and Sandberg, 2009). At the individual level, concerns are raised about medical safety and side effects, the authenticity of the enhanced mind and the value of achievements facilitated by pharmaceutical intervention. At the societal level, ethical questions can be asked about whether the availability of PCEs would increase or undermine equality, and about whether individuals will be directly or indirectly coerced into using PCEs. Further normative questions emerge particularly in the healthcare setting: should we be drawing a sharp line between treatment and enhancement and should individuals be given access to PCEs through medical professionals?

In this paper, we outline the key issues at stake in the normative debate about pharmacological cognitive enhancement (PCE) and, for each issue, suggest the contribution that neuroscientific research could make. The greatest contribution will be made to the discussions surrounding the safety and efficacy of PCEs. Although the question of what harms are worth risking in the pursuit of certain benefits is to a large extent normative, the dearth of evidence about the effectiveness and safety of PCEs in real-world contexts renders the discussion mostly hypothetical at this point. More research on the risks of dependency is also urgently needed. Data of this kind will be crucial for discussions about regulation, and for debates about the permissibility of requiring or encouraging people to use PCEs.

In addition to the contribution neuroscience will make to understanding the risk-benefit profiles of PCEs, we suggest that a more nuanced understanding of the neural systems affected by different substances will enrich the debate about whether PCE use constitutes cheating. Also related to cheating, we further suggest that the neuroscientific evidence on the functional trade-offs precipitated by some PCE adds an important dimension to the debate about whether achievements facilitated by PCEs should be seen to be effortless and involve little sacrifice. Drawing together our conclusions, we propose an “ethical agenda” for future neuroscientific research on PCE. This agenda sets out what sort of research would help move the ethical debates forward, and why. Resolving these debates will be crucial for ensuring that society responds to the increasing use of PCE in the most responsible, fair and rational way. For a summary of our “ethical agenda” for neuroscientific research, see Table 1.

**Table 1:** **Summary of ethical agenda for neuroscientific research**.

**Suggested type of study**	**Advancement in ethical debate**
Longitudinal studies investigating the long-term safety profile of PCEs	This is perhaps the most pressing task for neuroscientists. The long-term, real-world safety profile of PCEs is of considerable import to potential users and to all debates about PCE ethics and policy. In relation to the latter concerns, longitudinal studies will advance ethical debates about: (1) whether PCEs should be placed on the open market for enhancement purposes (and with what restrictions), and (2) whether employees doing particular types of jobs can legitimately be required to take PCEs
Identification of pathology associated with mental or psychiatric disorders or limitations to enable classificatory separation of conditions which are diseases from those which constitute normal human variation	Will advance the ethical debate about whether the administration and effects of particular PCEs constitute treatment or enhancement, and how resources should be deployed accordingly
Identification of the effects of PCEs in targeted and specified populations of ethical significance, such as those who are worst off. In particular, further research into the baseline effect should be conducted	Will advance the debate about distributive justice and access to PCEs. If PCEs have differential effects on those who are already worst off, this will be highly relevant to their permissibility and just distribution
More precise distinction between the different cognitive effects of different PCEs	Will (1) be of central relevance to whether certain putative PCEs will be used for enhancement and, if so, in which contexts and (2) advance the debate on cheating in competitive contexts: some effects (e.g., creativity) might be considered more unfair than others (e.g., wakefulness) and enhancing motivation vs. enhancing effectiveness might be considered relevant to the value of any resulting achievements
Investigation of the functional trade-offs associated with different PCEs	Will (1) be of central relevance to whether certain putative PCEs will be used for enhancement and, if so, in which contexts and will (2) advance the debate about the nature of the sacrifice possibly required for achievements to have value. It will also (3) advance the debate about the practicality and legitimacy of requiring certain people to take PCEs
Pursuit of a “personalized enhancement” approach to bring us closer to understanding what effect any particular PCE will have in any particular person	Will be relevant to many (if not all) ethical debates and policy considerations including: (1) whether particular people could legitimately be required to take PCEs in certain contexts, (2) who should be given priority access to which PCEs, (3) whether unequal effects have ramifications for cheating. Only when we can predict the *personal* benefits and costs of enhancement can policy be truly informed and ethical

## Overview of pharmacological cognitive enhancement

What it means to “enhance” is notoriously difficult to pin down. To enhance is essentially to improve or increase, but what this improvement must be relative to is not obvious. On the broadest definitions of enhancement, some capacity is enhanced if it is improved relative to its prior level of functioning such that it increases the individual's chances of leading a good life—enhancement thus occurs regardless of how well- or poorly-functioning the capacity originally was (Savulescu et al., 2011). On more restrictive definitions of enhancement, a capacity is enhanced if it is improved beyond a particular point—perhaps a species mean or agreed “normal” level of functioning (c.f. Sabin and Daniels, 1994). Others define enhancement as any improvement which goes beyond correcting pathology. For example: “A cognitively enhanced person [… ] is not necessarily somebody with particularly high (let alone super-human) cognitive capacities. A cognitively enhanced person, rather, is somebody who has benefited from an intervention that improves the performance of some cognitive subsystem without correcting some specific, identifiable pathology or dysfunction of that subsystem” (Bostrom and Sandberg, 2009). In this paper, we adopt the broader understanding of cognitive enhancement. We do this in part because the substances currently available and likely to be available in the near future effect only modest improvements (Husain and Mehta, 2011), but also because we believe that any line intended to mark the point at which an improvement counts as enhancement necessarily involves a value judgement involving normative (ethical) considerations.

Most of the substances cited as putative PCEs were originally developed for clinical use, to treat conditions that are at least partly characterized by some observable cognitive defect. Here, again, it is sometimes difficult to decide what should count as a cognitive *defect*. However, in the case of defective or deficient capacities, decisions must be made about where to place the line to determine who should receive medical attention and resources. For example, two of the substances receiving the most attention from those interested in enhancement—methylphenidate and modafinil—were originally developed to treat the symptoms of ADHD and narcolepsy, respectively. More recently, however, these substances have been used off-label by healthy individuals to improve their memories, level of alertness, or powers of concentration (e.g., Maher, 2008). Other substances with some modest enhancing effects on cognition include donepezil, dopamine agonists (such as d-amphetamine, bromocriptine, and pergolide), guanfacine, atomoxetine, reboxetine, galantamine, rivastigmine, and memantine. Working pharmacologically in different ways, these substances have been shown to improve cognitive functions such as response inhibition, working memory, episodic memory, attention, vigilance, and incidental learning (see de Jongh et al., 2008; Lanni et al., 2008; Husain and Mehta, 2011). However, this limited evidence of effectiveness should be cautiously considered alongside studies producing null results and some evidence of task-specific impairments (see Hall and Lucke, 2010 and Advokat, 2010 for less optimistic reviews of the scientific literature on PCE).

The prospect of being able to enhance any of these cognitive functions probably would be attractive to many individuals. Whether the goal of such enhancement would be to perform better at work, to learn a skill or language quicker, to decrease the need for rest in leisure time, or even just to experience one's mind as “sharper,” improving cognition would presumably come with many benefits. Data from various prevalence studies indicate that there are groups of individuals who use some of the substances listed above for purposes of studying, to combat jet-lag or even to facilitate completion of household chores (for a review of student uses, see Smith and Farah, 2011; see also Maher, 2008).

Whilst the neuroscientific literature is reporting some modest enhancement effects of these substances on the cognition of healthy individuals (c.f. Husain and Mehta, 2011), the ethical literature has been raising and responding to a variety of issues pertaining to their use (for overview see Greely et al., 2008; Bostrom and Sandberg, 2009). Some of these issues are practical, some socio-political and others relate to the individual user. The overarching goal is to ascertain how permissible and how moral PCE use is and how society and regulatory bodies should respond to it. Although the ethical debate is principally a normative enterprise, it cannot reach firm conclusions about how to proceed based purely on hypothetical reasoning and untutored speculation: it must be informed by neuroscientific research providing the empirical facts about PCEs. In what follows, we outline the key issues in the enhancement debate, emphasizing where we think neuroscientific research might have particular importance for the normative debate.

## Ethical debate and the relevance of neuroscientific research

### Medical safety and effectiveness

In many ethical discussions of cognitive enhancers the first issue to be raised (often to be set aside so that there can be any further discussion at all) is whether cognitive enhancers are *medically safe* to use. Since there are no longitudinal studies yet examining the long-term use of pharmaceuticals such as modafinil and methylphenidate, some authors argue that we currently do not know enough about the potential dangers and that the availability and use of PCEs should be avoided on this basis (e.g., Drabiak-Syed, 2011; Boot et al., 2012).

Despite the huge interest in PCE from philosophers and scientists, the evidence of their *effectiveness* is still inconclusive. Moreover, where there is evidence of enhancement effects, they often tend to be limited to improvements on specific tasks, are only seen at certain dosages and are not observed in all people (Ragan et al., 2013; Farah et al., 2014). Crucially, it must be remembered that the degree and nature of any cognitive improvements will be different for each PCE and so no sweeping claims should be made about the effectiveness of PCEs in general. In terms of both effectiveness and safety, it should also be noted that short term studies carried out in laboratory settings are not representative of long term use in real world contexts.

In his meta-analysis of randomized controlled trials of methyphendidate, Repantis et al. (2010) found a significant improvement in the long-term memory of healthy participants, particularly when there was a longer interval between the learning phase and recall. However, the meta-analysis revealed no significant improvements in attention, mood or executive functions. Similar findings emerged from Farah et al.'s (2014) review of more than fifty experiments on the effects of amphetamine and methylphenidate: they found convincing evidence of an enhancing effect of stimulants on learning under some circumstances, specifically when the retention interval between study and test was longer than an hour, but not at shorter intervals. They also concluded that the evidence for improvement of executive functions was much less clear. There is some evidence to suggest that the effects of methylphenidate on cognitive control are only significantly positive in participants whose performance on placebo was lowest (Smith and Farah, 2011).

In relation to the effectiveness of modafinil, Farah et al.'s (2014) recent review of single dose studies of modafinil concluded that there is clear evidence of enhanced executive function and memory for sleep-deprived individuals but, for rested adults, whilst there were some positive findings for specific tasks such as those requiring inhibitory control, there were also a large number of null results and the occasional finding of impairment. They refer to this pattern—of limited improvements on some specific tasks and impairment on others—as being “familiar” for PCEs.

There are also some reviews of the effectiveness of anti-dementia medications for cognitive enhancement. These include acetylcholinesterase inhibitors such as donepezil, rivastigmine, and galantamine. A review conducted by Repantis (2013) concluded that the few existing studies of effects in healthy participants provide no consistent evidence for a neuroenhancement effect. In the case of, donepezil there was some evidence to suggest improvements on retention of training on complex aviation tasks (Yesavage et al., 2002), improvements in verbal memory and episodic memory (Gron et al., 2005). However, other studies showed no or limited effects on memory and attention and two others showed transient impairment of episodic memory (Beglinger et al., 2004, 2005). The same pattern of results suggesting enhancement in some cases but no effect or even impairment in others can be seen for donepezil. Further, a review of the efficacy of these putative cognitive enhancers for patients with mild cognitive impairment concluded that they did not improve cognition or function among patients with low-level impairment (Tricco et al., 2013).

The *medical safety* of PCEs varies from substance to substance, and side effects relate not only to the direct pharmacological effects but also to broader psychological and physiological changes. The review conducted by Repantis (2013) concluded that in the majority of trials, the drugs were well tolerated. However, side effects were noted. In relation to methylphenidate, side effects included increased heart rate and some instances of increases in blood pressure. Headaches, anxiety, nervousness, dizziness, drowsiness, and insomnia were also typical complaints.

Repantis (2013) summarizes similar side effects for modafinil, where adverse reactions included headache, dizziness, gastrointestinal complaints (e.g., nausea, abdominal pain, dry mouth), increased diuresis, palpitations, nervousness, restlessness, and sleep disturbances and insomnia (especially in studies with non-sleep deprived individuals). In their recent review, Ragan et al. (2013) highlight the fact that modafinil was reviewed by the European Medicines Agency (2010), who concluded that it should not be prescribed for obstructive sleep apnea, shift-work sleep disorder, and idiopathic hypersomnia because of the risks of serious skin reaction, suicidality, depression, psychosis, and adverse cardiovascular events.

In relation to anti-dementia drugs, Repantis (2013) concluded that, in the majority of the trials in healthy adults, donepezil was well tolerated. However, some side effects were reported in some participants, including gastrointestinal complaints (e.g., nausea), headaches, dizziness, nightmares, and insomnia. The meta analysis of anti-dementia drugs for people with mild cognitive impairment (Tricco et al., 2013) revealed that patients taking these medications experienced significantly more nausea, diarrhea, vomiting, and headaches than patients taking placebo. The authors also suggest that patients taking these medications might be at greater cardiac risk, with one study finding a higher incidence of bradycardia among patients who received galantamine.

As Farah et al. (2014) emphasize, there is another type of risk that should not be ignored in a consideration of the safety of PCEs. Many pharmaceuticals, especially stimulants, present a risk of dependence. The authors cite a nationwide survey analyzed by Kroutil et al. (2006) which estimates that almost one in twenty nonmedical users of prescription stimulants meet the criteria for dependence or abuse (For further discussion of the potential for addiction in student populations see Outram, 2010 and White et al., 2006).

Finally, as Ragan et al. (2013) point out, there is no such thing as a completely safe drug, only a drug whose benefits outweigh its drawbacks. However, it is also worth emphasizing that, even if there are long-term risks associated with these substances, this does not (by itself) mean that they should automatically be prohibited. There are serious risks associated with many activities that the state permits because it is believed that individuals should decide for themselves whether these risks are worth taking. Dangerous sports and cosmetic surgery both come with risks, but the value some individuals attach to the respective sporting experiences and cosmetic effects justifies giving these individuals the choice to take risks in their pursuit.

This caveat notwithstanding, and taking into account potential costs to the healthcare system, greater knowledge about safety and efficacy will allow regulators to decide whether the decision about which risks are worth taking should be put in the hands of consumers (for a detailed discussion of the way risks and benefits should be assessed for cognitive enhancement devices, such as brain stimulators, see Maslen et al., 2014). The ethical debate about the level of risk consumers should be allowed to take is of great practical importance when it comes to making policy recommendations. In addition, the question of whether the harms of a certain PCE outweigh its benefits will be important to discussions about the permissibility of requiring individuals to use PCEs and about the possible need to protect individuals from pressure to take any of the substances under discussion.

Finally, the empirical project of identifying the different effects PCEs have across a different individuals (c.f. Husain and Mehta, 2011) is likely to feed into the normative debate about which effects (for which individuals) constitute a form of treatment and which effects (for which individuals) constitute enhancement. We discuss these and other ethical issues in what follows.

### Authenticity and naturalness

There are a bundle of related ethical issues that are sometimes raised under the broad heading of *authenticity* (see Bublitz and Merkel, 2009; Juth, 2011). Some of these pertain to numerical personal identity—do individuals become categorically different persons when they transform themselves via enhancement? (DeGrazia, 2005)—some consider less drastically what it is for an individual to be to be more or less his or her “real” self (The President's Council on Bioethics, 2003), and other ethical concerns pertain to what it is to be, and function as, a human being (Kass, 2003).

The principal tenet underlying authenticity objections against the use of PCEs is that individuals are most themselves when they are in their “natural,” unaltered state. If capacities and characteristics fundamental to one's identity are changed, then the individual is recast as an altered or inauthentic person (e.g., Elliott, 1999). This argument is premised on the idea that there is a “real,” true self, and that this real self is to be preserved as much as possible. However, this assumption can be challenged: individuals often (and understandably) try to improve themselves in ways that allow them to more successfully achieve their goals. Being autonomous is to form goals for how one's life is to go, including what kind of person to be. On this model of authenticity as autonomy, whether PCE is authentic depends on whether it helps a person to achieve her autonomous goals. For example, an individual might teach him or herself motivational strategies to overcome his or her naturally lazy disposition; another individual might use techniques from cognitive behavior therapy to overcome his or her propensity for generalized anxiety (e.g., Butler et al., 2006) or shyness, or gregariousness, or bad temper, or gullibility. Such strategies may not render the individuals inauthentic, but rather assist them in removing barriers that otherwise prevent them from maximizing self-actualization. Correspondingly, if PCEs can, for example, help an individual to concentrate better so that he or she can achieve the goals he or she values, this acts in service of authenticity rather than undermines it. There is great human variation, and variation within individuals subject to many intrinsic and extrinsic factors (see Kahane and Savulescu, 2013). Even if the authentic self were defined, it seems likely that many factors interfere and PCEs may reduce the effect of such influences.

However, some deny that authenticity is reducible to autonomy. Such writers (e.g., Taylor, 1991) appeal to a “real self.” But even on such an account, the real self may be complex and multifaceted. Often people have a range of qualities and they may use PCEs to bring out some of their qualities, while suppressing others. Thus, whether an enhanced self compromises the real self depends on what constitutes a person's real self and what the effect of the PCE is—both questions for cognitive science. If PCEs merely amplify, rather than add entirely new qualities, then they enable the self to evolve, rather than replacing one individual with a set of attributes with another with different attributes.

There is a related but different concern about *naturalness*. The idea that enhancements will take us too far from what it is to be human altogether is often accompanied by the idea that too much technological intervention will lead to an over-mechanization of the mind. The activities in which we engage—and, more importantly, the ways in which we engage in them—are said to have a certain quality to them that makes them “human” activities (President's Council on Bioethics, 2003). In this vein, Kass (2003) argues that since individuals play no role in bringing about the effects of biomedical interventions, they cannot understand these effects “in human terms.” His suggestion is that whereas the effects of studying or training are “intelligible” to us, the effects of direct interventions are not comprehensible and thus our use of them departs “from “genuine,” unmediated, and (in principle) self-transparent human activity” (p. 23).

However, we argue that we make use of many directly-acting substances, in medicine and in leisure, that do not result in departure from “genuine” human activity. Just because their pharmacological mechanisms are not understood by the average person does not mean that they cannot be made sense of as part of a human narrative. Kass cites alcohol, caffeine and nicotine as not having the same unintelligible quality as direct biomedical interventions. He says this is because “we use these agents not as pure chemicals but in forms and social contexts that, arguably, give them a meaning different from what they would have were we to take them as pills” (p. 22). An obvious objection to Kass' resistance to PCEs would be to add PCEs to beverages, as caffeine currently is. It would then be “intelligible” in the same way that caffeine is said to be “intelligible.” Moreover, if intelligibility can be conferred by social context then the social context of, for example, studying, or conducting research should equally make PCEs part of a comprehensible human enterprise. Perhaps his distinction between the forms alcohol, caffeine, and nicotine tend to take, and the form of a simple pill, is supposed to indicate that the former are enjoyed for themselves, rather than being instrumental to achieve some goal. However, studies have reported that some individuals take PCEs for recreational purposes (see Smith and Farah, 2011) and it is common knowledge that caffeine is regularly used exclusively for alertness and for performance enhancement. Even if it might be the case in lay people's current perceptions (cf. Faulmüller et al., 2013; Schelle et al., 2014), from a normative stance it cannot be that form and context make all the difference between the human intelligibility of an espresso and a caffeine pill and a PCE.

The core of such an “intelligibility” objection may be that PCEs and other new technologies work in ways entirely alien to the way the human mind normally works, adding a completely new way of being. For example, chips inserted into the human brain that allowed us to perceive other people's thoughts directly would be entirely new. Neuroscience can assist by unravelling the way the mind does work, and does not, and by enabling categorization of enhancers into those which harness natural processes, and those that introduce entirely new capacities. Most enhancers at present appear to harness existing neurobiological physiology, though exactly how many enhance performance remains to be determined.

The ethical debate about authenticity and naturalness is unlikely to be advanced solely by the findings of neuroscientific research. The disagreement is partly a normative one about what constitutes the “real” self and whether our “real” selves are the selves we are most prone to being or the selves that we aspire to develop in to—or whether it makes sense to speak of “real” selves at all. Qualitative research, such as that conducted by Singh (2005) or Bolt and Schermer (2009), will helpfully provide a clearer picture of the sorts of experiences individuals have when taking PCEs.

In summary, it is important to recognize that most PCEs, if not all, harness innate biological systems, for example, affecting release, reuptake or sensitivity to neurotransmitters that cause cognitive activity. They do not at present introduce radical “new ways of being” divorced from the ordinary human way—they really just provide “more of the same.” Indeed, humans vary in the ways in which their cognitive systems function and in some cases, PCEs may bring those at the lower end of normal up to the level of function of those in the mid to upper range.

More importantly, we suggest that what matters more than whether the experiences are in some sense authentic is whether the individual wants and values the effects of the PCE and whether the individual is autonomous in his or her decision to use PCE. This, we suggest, is a legitimate concern and is addressed in the following section.

### Coercion

If PCEs were to become more commonplace, then employers might start to require their employees to use PCEs. The Academy of Medical Sciences et al. (2012) suggested in a recent report that “[O]ccupations that require particular patterns of focus could benefit from enhancements that facilitate achieving such patterns. For example, surgeons may need to be able to concentrate for extended periods, whereas other jobs such as air traffic control can require very rapid reactions during periods of relative uniformity. As an extrapolation to this, it is possible that in these high-responsibility occupations enhancement could be seen as a moral obligation, or even demanded by the public.” (p. 38, for a discussion see also Maslen et al., in press). The US Airforce has already approved the use of modafinil by its pilots (Caldwell and Caldwell, 2005) and some medical practitioners are beginning to wonder whether enhancement might be required of them in the future (Rose and Curry, 2010). Writing in the Journal of Surgical Research, surgeons have suggested that the use of PCEs may come to be required practice. They say, “The prospect of fatigued surgeons taking a prescription drug, such as modafinil, to allow them to operate for longer, and possibly to a higher standard, is perhaps not as far-fetched as some may suggest. This drug has already been trialed in emergency physicians, when performing non-medical-related tasks at the end of a nightshift.” (Warren et al., 2009, p. 168).

Further, the authors note that there are “useful and warranted forms of coercion” (p. 170) such as forcing surgeons to undertake hygiene practices such as handwashing prior to and during surgery. Given that this *coercion* is acceptable, they go on to ask, “What will our employers feel about a drug that makes us less prone to error, able to work longer hours, or to operate more efficiently? Employers are able to request certain behavioral standards from their employees, dictate rest periods, and insist on abstinence from certain drugs to ensure that their doctors perform well—will a day arise where they can recommend or even insist on surgeons being artificially enhanced? This may seem fanciful, but recent work has suggested that a mixture of napping and caffeine attenuates fatigue in interns and thus should be adopted by hospital administration. Why not other types of stimulant?” (p. 171).

The ethical objection often raised in this context is that, although it is thought to be reasonable to require certain things of employees, such as compulsory training and codes of conduct, requiring them to ingest psychoactive substances into their bodies is too demanding a requirement. It would require a compelling justification (perhaps pointing to the severity of harm that would be prevented through requiring enhancement) to trump the value we place on preserving the right individuals have to determine what happens to their bodies and minds (for discussion of the right to mental self-determination in relation to enhancement and other mental manipulation, see Bublitz and Merkel, 2014). As far as possible, this right should be preserved, and this is especially the case where there is not enough evidence about the harms to which an employer would be subjecting his or her employee. Neuroscientific evidence will have a large role to play in understanding the seriousness of any proposed requirement. In addition to the risks posed by individual instances of PCE use, more data on the potential for dependency will be essential for this discussion. Whilst we *might* think it permissible to require some employees to take small, isolated personal risks, requiring them to do something that results in substance dependency would more comprehensively infringe an individual's autonomy. In this connection, although PCEs may become more common in the workplace, one of us has argued elsewhere that for these and other reasons, it is unlikely that there will ever be a legal obligation for a professional like a surgeon to take a PCE (Goold and Maslen, 2014). At present, no employer requires employees to take caffeine. Caffeine is a PCE.

Even if people were not directly coerced to take enhancers it could still be objected that permitting PCE use could result in indirect pressure to use them. The perception that others are taking substances that make them more productive could lead to the belief that taking them is necessary to keep up (Academy of Medical Sciences et al., 2012) and not taking PCEs might render one *de facto* ineligible for certain jobs (Chatterjee, 2004). However, whether indirect pressure to take PCEs would in fact result in their more prevalent use is a question for social science. (For empirical data relating to this question, see Franke et al., 2011 and Maier et al., 2013). Neuroscientific research will have little to contribute to the debate about the limits of acceptable social pressure and restriction on employees' autonomy. However, as noted above, opposition to enforced PCE use is partly motivated by the current lack of evidence on long-term safety and efficacy. What we can legitimately require of people is closely related to what risks we can require them to take. Assessment of the legitimacy of requiring certain individuals to take PCEs will depend in large part on their medical safety and efficacy. If PCEs are very safe and efficacious, their use in life-saving/threatening professions (e.g., surgeons, politicians, truck drivers, airline pilots, etc.) may legitimately be required.

### Treatment vs. enhancement

As noted in the introductory section, there is much disagreement about what should count as enhancement (c.f., Parens, 1998). Sometimes this disagreement is framed as a debate about where *treatment* ends and *enhancement* begins. The distinction often made is that treatments serve to cure illness and preserve health whereas enhancements make people “better than well.” For example, Juengst (1998) defines enhancement as the term “usually used in bioethics to characterize interventions designed to improve human form or functioning beyond what is necessary to sustain or restore good health” (p. 29).

However, a common objection to this distinction is that, in many cases, what we define as “healthy” and “normal” is arbitrary. This objection does not deny that there can be clear failures of function or physiology as a result of pathology which most would agree are inimical to good health, such as the effects of a brain hemorrhage or stroke. Rather, it emphasizes that the boundary between healthy and unhealthy cognition in many cases is a matter of where we choose to draw the line, not based on either statistically significant subfunctioning or pathology. For example, delimiting normal from defective powers of concentration when diagnosing ADHD is necessarily to engage in marking a categorical point on what is otherwise a continuum (c.f. Schermer and Bolt, 2011). The point could be selected further to the left or right on that continuum of functioning. Would selecting a point which increased ADHD diagnosis increase the instances of individuals being treated or would some be receiving enhancement through the back door? Since the point is to some extent arbitrary, the corresponding labels of treatment and enhancement appear less meaningful in this context.

Similarly, it is difficult to know whether to classify substances used to combat age-related cognitive decline as instances of treatment or enhancement. Drawing sharp lines could have the result that a young person with cognitive abilities just above the cut off for being classified as having a mental disability would be “enhanced” by a drug but the elderly person whose abilities slipped to a level still above the young person would be receiving “treatment” if given the same substance (for a similar example, see Sandberg, 2011). Given the slipperiness of the distinction, one of us has argued (Savulescu et al., 2011) that instead of trying to determine whether certain drugs or certain of their effects constitute treatment or enhancement, it is more coherent and useful to think of a continuum of well-being which can be increased or diminished by various interventions.

It might be thought that evidence from neuroscience could adjudicate between instances of treatment and enhancement. If substances have discernable, discrete effects on different groups of people, it could be argued that these discrete effects mark the difference between a treatment and an enhancement. For example, although the way modafinil works is still unknown in detail (Minzenberg and Carter, 2008), neurologists do know that the brain of the narcoleptic is not neurophysiologically equivalent to the brain of the sleep-deprived individual and, correspondingly, it might be hypothesized that the effects of modafinil on the two groups will differ. Most forms of narcolepsy are associated with a deficiency in the hypothalamic neurotransmitter orexin (Mignot, 2010). The average sleep-deprived person, in contrast, does not exhibit such a deficiency. Accordingly, it might be thought that the more differences neuroscience can reveal between the narcoleptic and the non-narcoleptic, the better equipped we will be to distinguish between the treatment and enhancement effects of at least this PCE.

However, such knowledge would still not provide a definitive solution to which effects we should refer to as treatment and which we should call enhancement. Modafinil is also prescribed for shift work sleep disorder (SWSD), which is a product of unusual working patterns affecting circadian rhythms, not of underlying neurophysiology (Åkerstedt and Wright, 2009). This being said, it should be noted that not everyone who does shift work suffers from SWSD. This suggests that there must be some physiological or psychological difference between sufferers and non-sufferers and our lack of knowledge as to the cause of this difference does not make the disorder less of a treatable disorder.

In labeling the prescription of PCEs for SWSD an instance of treatment, a normative or ethical decision is still being made about which conditions and patterns of functionality should attract medical attention and resources. We are also implicitly making an assessment that medical treatment is the just and appropriate course of action for sufferers of the disorder, rather than prioritizing a change away from shift work. Neither the individual's underlying neurophysiology nor the particular mechanism of action of the substance tells us anything about whether this decision is the correct one.

One avenue through which neuroscience might illuminate the treatment vs. enhancement debate is by identifying pathology associated with mental or psychiatric disorders or limitations. So far, accurate tissue or cellular level pathological classification of psychiatric disease or disorder has eluded researchers. However, if psychiatric disorders could be characterized in the same way as neurological disorders, the presence of pathology would separate conditions which are diseases from those which constitute normal human variation.

Given that PCEs are not universally available through the healthcare system, individuals without conditions for which PCEs are approved would currently have to obtain them through other, unauthorized routes. This means that some people will have access to them but others will not. Even if PCEs were available on an open market, there could still be financial or other barriers to their accessibility. We discuss this issue and its potential implications next.

### Distributive justice

Society-level debates about PCE-related inequality consider *distributive justice*, and are related the question of whether PCEs will exacerbate existing socio-economic inequality. A common argument is that, as with many technologies, the rich and informed will have access to them whilst the poor and uninformed will not (e.g., Fukuyama, 2002). Assuming that cognitive enhancement confers some benefits, this will make those already at an advantage even better off. Whether this would in fact happen would depend on factors such as the affordability and accessibility of PCEs, as well as on the realities of their cognition-improving effects: the affordability and accessibility of PCEs will determine whether people are able to use them; the effects of the substances will determine whether they really put people who do so at an advantage. However, although there is the potential for PCEs to exacerbate unfairness if their distribution is unregulated, as one of us points out elsewhere, this is not a necessary consequence (Sandberg and Savulescu, 2011): if PCEs were distributed according to a principle of justice such as “prioritarianism”—the principle that says that we should give priority to those who are worst off, but also aim to maximize well-being of everyone in society—then PCEs would be most accessible to the worst off, becoming less accessible (but not inaccessible) as need decreases.

Further as we go on to discuss below in relation to competitive fairness, neuroscientific evidence supports the hypothesis that there is a base-line effect of many PCEs: their effects seem to depend on the subject's baseline working memory capacity. Individuals with low working-memory capacity improve while high-span individuals are either not affected or are even impaired (de Jongh et al., 2008). This means that those most in need of PCE would benefit most from it, with those less in need not benefiting at all or even experiencing impairment from the same substance. Given this evidence, it has been suggested that enhancement might actually serve to *reduce* inequality (Bostrom and Sandberg, 2009). However, whilst this could be true in terms of the equality of cognitive capacity, it must be remembered that cognitive capacity and socio-economic status are not always correlated: there would still be people with more opportunities and resources who could improve their prospects further. Whilst policy decisions about access to PCEs will be principally socio-political matters, those making the decisions will need to know how enhancers affect members of the population in order to best serve the interests of justice and equality. If PCEs have differential effects on those who are already worst off, this will be highly relevant to their permissibility and just distribution. Neuroscience research can thus contribute to ethical debate if effects in targeted and specified populations of ethical significance are studied. This would require ethically relevant population stratification.

### Competitive fairness and cheating

The ethical discussion of whether using cognitive enhancers constitutes *cheating*—perhaps in exams or at work—is more nuanced than the simple question of whether taking enhancers is “against the rules.” It can extend beyond considerations of *fairness in competitive contexts* to ask whether personal achievements facilitated by PCEs are devalued for this reason (c.f., Schermer, 2008; Goodman, 2010; Santoni de Sio et al., in press). We suggest that evidence from neuroscience will help to develop the cheating debate in important ways. Below, we argue that three types of empirical inquiry are relevant to the ethical discussion. The first, the phenomenon of the “inverted U”—according to which the enhancing effects of PCEs are often baseline dependent and exhibit non-linear dose response curves —(de Jongh et al., 2008; Husain and Mehta, 2011), is relevant to efficacy questions involved in debates about cheating. The second type of study relevant to the debate is that which seeks to identify the particular neural systems affected by different substances, leading to disparate effects (e.g., Lanni et al., 2008): whether a substance improves creativity or rote learning may matter for some possible conceptions of what constitutes cheating. Similarly, whether a substance improves motivation and task enjoyment vs. memory capacity might matter for those who place a lot of value on success requiring effort. Third, we argue that the neuroscientific evidence pointing to the likelihood of cognitive trade-offs (de Jongh et al., 2008; Husain and Mehta, 2011) adds an underdeveloped dimension to the cheating debate: if the complaint is that achievements facilitated by PCE are devalued because they do not involve enough personal sacrifice, then evidence suggesting that enhancement in some domains comes at the cost of impairments in others offers a challenge to this view.

#### The inverted U curve and baseline dependency

Neuroscientific research so far shows that the effects of many purported PCEs are base-line dependent and have an inverted U-shaped dose-response curve (de Jongh et al., 2008; Husain and Mehta, 2011). This is important to the cheating debate as it means that some individuals will benefit from taking PCEs whereas others will gain no benefit and might even be impaired: low performing individuals will tend to be on the upward slope of the inverted-U and so benefit from a substance that moves them further up this slope. High performing individuals, on the other hand, will tend to be at the peak of the inverted U and will therefore become impaired by a substance that increases neurotransmitter levels further. If neuroscience were to more precisely identify the neurological profiles of those who are able to benefit from PCEs and those who are not then ethicists would be able to consider in greater detail whether the prospect of some being able to enhance whilst others cannot counts more decisively against PCE in competitive contexts than if all could enhance in these contexts. They would need to consider whether it is the case that enhancement is only fair if everyone could (in principle) avail themselves of it or whether is it permissible given that some are physiologically denied the possibility of improving.

#### Disparate effects of different PCEs

Although the exact mechanisms of substances like methylphenidate and modafinil are not yet fully understood, researchers have begun to investigate which PCEs affect which underlying systems, and with which effects (Lanni et al., 2008; Smith and Farah, 2011). Although cognitive functions necessarily interact, attempts have been made to ascertain the primary cognitive functions improved by particular PCEs based on their effects on neurotransmitters. Husain and Mehta (2011) explain that “a simple mapping between a specific neurotransmitter and a particular cognitive function—such as [working memory]—[… ] seems untenable. However, subtle but important differences in the precise processes modulated might provide some discriminating value: for instance, dopamine has an established role in reinforcement learning in response to rewards, whereas serotonin seems to modulate reinforcement learning for aversive stimuli.” (p. 29). Pursuing such discrimination, Lanni et al. (2008) review the neuroscience literature investigating the neuronal circuits, neurotransmitters and molecular events underlying the cognitive domains of memory, attention, and creativity to distinguish the effects of different enhancement substances. Elsewhere, Smith and Farah (2011) review the cognitive neuroscience literature to examine whether (and which) prescription stimulants improve learning, working memory, cognitive control, and other executive functions.

If neuroscientific research were able to distinguish between the effects of different PCEs this could have some implications for discussions about cheating. This is again effect stratification. Combined with population stratification, neuroscience research could bring us closer to understanding what effect this particular PCE will have in this person. This reflects the move to “personalized medicine” and might be dubbed “personalized enhancement.” Only when we can predict the *personal* benefits and costs of enhancement can policy be truly informed and ethical.

It might be thought that the enhancement of some cognitive functions is more unfair than the enhancement of others. For example, the enhancement of creative thinking might be thought to constitute more significant cheating than improving wakefulness or even memory capacity. Imagine someone who says “when I take enhancers my work is no better, I can just do more of the same for longer” vs. someone who says “when I taken enhancers my work is much better than I can do without them.” Having links with the debate about authenticity, it is as if the former individual is enabled to make better use of his or her own cognitive resources, whereas the latter is given new cognitive resources upon which he or she can draw. Those who think PCE use is unfair because the achievement is not a reflection of the person's natural abilities to solve and create might be less concerned by a PCE that simply allowed more efficient work of the standard the person could naturally achieve. A PCE that promoted wakefulness might allow an individual to work for longer but it will not come up with ideas on his or her behalf. Of course, it is important to remember that a PCE that improved creativity still has its effects on and through the individual's own brain. What will be interesting for ethicists to discuss is whether “assistance” with time management and efficiency is relevantly different to “assistance” with the content of ideas (if, indeed, we want to characterize the respective effects in this way).

Practical consequences might be to consider certain substances unfair for certain types of tests or for entry into certain types of employment: employers might only be troubled by the use of PCEs, the effects of which are *necessary* to carry out the job. This would be a practical consideration: could the employee continue to work without the PCE? For example, an architect who could only perform satisfactorily when taking a substance like modafinil that seems to improves spatial planning and visual pattern recognition memory (Turner et al., 2003) might be thought to be a higher risk employee than one who uses a memory enhancer which enables him or her to remember the names of building materials that he could look up without problem in the absence of the substance.

Further, neuroscientific research that could distinguish between substances that enhance the *effectiveness* of cognitive capacities, such as working memory, from those that instead (or additionally) increase *motivation* could also have implications for the competitive fairness debate. In the ethical literature, the point is sometimes made that it is effort and striving that makes achievements intelligible and valuable. For example, Fox (2005) argues that “[b]ecause they act directly on the human body and mind, biotechnological enhancements tempt us to shirk individual striving and struggle” (p. 1150).

A common rebuttal to this type of argument is that, whilst PCEs can make efforts more effective, they do not replace the need for dedicated, sustained study—striving and struggle is still required in order to achieve. For example, Greely (2010) notes that “the more plausible cognitive enhancements would not eliminate the need to study; they would just make studying more effective” (p. 6).

If, however, there were a significant enough effect of a PCE on motivation and/or task enjoyment, then it would be open to ethicists to argue that this *does* in some sense reduce the amount of effort that the person puts in. The drive to work or achieve no longer emanates from the individual and no struggle is encountered.

On the motivating effects of prescription stimulants, Smith and Farah (2011) write: “Another empirical question concerns the effects of stimulants on motivation, which can affect academic and occupational performance independent of cognitive ability. Volkow et al. (2004) showed that [methylphenidate] increased participants' self-rated interest in a relatively dull mathematical task. This is consistent with student reports that prescription stimulants make schoolwork seem more interesting (e.g., DeSantis et al., 2008). To what extent are the motivational effects of prescription stimulants distinct from their cognitive effects, and to what extent might they be more robust to differences in individual traits, dosage and task? Are the motivational effects of stimulants responsible for their usefulness when taken by normal healthy individuals for cognitive enhancement?” (p. 735).

If particular PCEs were shown to significantly improve motivation and/or task enjoyment whilst others only improve effectiveness, ethicists would need to consider whether there is any relevant difference between enhancing motivation and enhancing effectiveness and, if so, what the implications would be for the value of resulting achievements.

#### Enhancement is likely to involve trade-offs

Research suggests that enhancing one domain of cognition might come at the cost of impairing another. de Jongh et al. (2008) review evidence suggesting trade-offs between long-term memory and working memory; between stability and flexibility of long-term memory; between stability and flexibility of working memory; and perhaps, they conjecture, between cognition and mood. If a PCE comes at a cost—and, especially, a mental cost—this could also add a new dimension to the debate about cheating and the value of achievements.

In terms of gaining an unfair advantage over others in exams and other competitive tasks, the trade-offs would be relevant if the test required exercise of *both* the enhanced and the impaired capacity. Whilst the individual gains some advantage in some parts of the test, he or she would be disadvantaged in other parts. More generally, neuroscientific evidence of trade-offs are interesting to the debate about fairness and the value of achievements because some of the objections rest heavily on the idea that using PCEs means that no sacrifice—usually conceived as sacrifice of time, energy or other opportunities—is made by the individual.

For example, Kass (2003) says: “Yet in those areas of human life in which excellence has until now been achieved only by discipline and effort, the attainment of those achievements by means of drugs, genetic engineering, or implanted devices looks to be “cheating” or “cheap.” We believe—or until only yesterday believed—that people should work hard for their achievements. “Nothing good comes easily.”” (p. 21).

If enhancement of one domain of cognition comes at the cost of another then it does seem that some sort of sacrifice has been made. We might conceive of an individual who chooses to enhance his or her working memory such that he or she can solve complicated puzzles quickly. This same individual might accept that this enhancement comes at the cost of him or her finding it harder to recall facts and experiences from longer ago. Accordingly, whilst the physical act of ingesting a substance might be easy, there is a sense in which the enhanced capacity did not come easily—it did not come without personal cost. Whilst the conceptually most interesting trade-offs will involve impairments to cognitive capacities—like for like—it should also be noted that the more general side effects of PCEs (discussed in relation to medical safety above) also constitute an additional sort of “cost” to enhancement. The evidence on medical safety reviewed in section Medical Safety and Effectiveness suggests that PCE use will always come at a cost and may involve multiple costs of different kinds. The number and nature of these unavoidable costs constitute further challenge to the view that achievements facilitated by enhancement involve no sacrifice.

Important to note is that these costs of a trade-off are not like financial costs, which can be trivial and will constitute diminishment only insofar as they prevent the individual from making other purchases important to him or her. Rather, the costs of an enhancement trade-off are often mental costs—like for like—and are of a kind much more likely to constitute diminishment. Thus, neuroscientific research poses questions for those engaged in the cheating debate about whether there are relevant differences between different various costs of achievement—effort, opportunity, physiological side effects, cognitive trade-offs—and which (if any) are required for achievements to involve a sufficient level of sacrifice.

## Conclusion

We have reviewed six of the main issues debated by ethicists working on PCE. Often, their purpose in debating these issues is to clarify concepts and normative positions, which then serve as a basis for recommending how society—and especially those tasked with its regulation—should respond to the emergence of PCEs. We have argued that whilst some of these issues are mostly political (coercion) or metaphysical (what constitutes authenticity), others have much to gain from emerging neuroscientific research. As well as providing data on safety and effectiveness, neuroscience will also allow a more fine-grained debate about whether the effects of some PCEs are more unfair than others in competitive contexts and whether employers should be more wary of employee reliance on some PCEs than on others. Further, due to emerging evidence on trade-offs, those who object to PCE on the ground that it facilitates individual gain without any attendant pain will have to explain why accepting an associated impairment in exchange for an enhancement is not a relevant sacrifice. Although we anticipate that ethicists will be far from stumped by this challenge, we hope to have demonstrated that it will, in large part, be though responding to emerging scientific evidence that normative accounts become more refined, complete and practically relevant.

In general, neuroscience can contribute to the formation of ethical policy on PCEs by adopting a “personalized” approach: personalized enhancement. Fine grained and stratified research should seek to identify specific risks, benefits, and trade-offs in small ethically relevant populations, or ideally in individuals. In doing this, according to the ethical values principles and criteria we choose, we can form policy on who should access which PCEs in which ways.

### Conflict of interest statement

The authors declare that the research was conducted in the absence of any commercial or financial relationships that could be construed as a potential conflict of interest.
